# Effect of Shenfu Injection on Reperfusion Injury in Patients Undergoing Primary Percutaneous Coronary Intervention for ST Segment Elevation Myocardial Infarction: A Pilot Randomized Clinical Trial

**DOI:** 10.3389/fcvm.2021.736526

**Published:** 2021-12-03

**Authors:** Xiao Wang, Huangtai Miao, Yan Yan, Ruifeng Guo, Wei Gong, Yi He, Hui Wang, Xinliang Ma, Shaoping Nie

**Affiliations:** ^1^Center for Coronary Artery Disease, Beijing Anzhen Hospital, Capital Medical University, Beijing, China; ^2^Department of Radiology, Beijing Anzhen Hospital, Capital Medical University, Beijing, China; ^3^Department of Radiology, Beijing Friendship Hospital, Capital Medical University, Beijing, China; ^4^Department of Emergency Medicine, Thomas Jefferson University, Philadelphia, PA, United States

**Keywords:** myocardial infarction, primary percutaneous coronary intervention, reperfusion injury, Shenfu injection, cardiac magnetic resonance imaging

## Abstract

**Background:** Shenfu injection is a traditional Chinese medicine formulation that alleviates ischemia-reperfusion injury through multiple pharmacologic effects. However, no data are available regarding its efficacy in patients with myocardial infarction. We aimed to examine the effects of Shenfu injection on infarct size in patients with ST segment elevation myocardial infarction (STEMI) undergoing primary percutaneous coronary intervention (PCI).

**Methods:** From April 2016 to February 2018, 40 patients with first-time anterior STEMI undergoing primary PCI within 6 h of symptom onset were randomized 1:1 to intravenous Shenfu injection (80 ml Shenfu injection + 70 ml 5% glucose injection) or placebo (150 ml 5% glucose injection) before reperfusion. Treatment started before PCI and maintained for 5 days after PCI. The primary end point was infarct size assessed by CK-MB area under the curve (AUC) over 72 h and cardiac magnetic resonance (CMR) imaging 4 ± 1 days after PCI.

**Results:** Infarct size by area under the curve for CK-MB over 72 h did not differ between the Shenfu injection and placebo groups (5602.5 [3539.4–7526.4] vs. 6403.2 [2234.4–8340.6] ng·h/ml, *P* = 0.82). Among 32 patients who underwent CMR Imaging, a nominal reduction in infarct size was observed in the Shenfu injection group compared with the placebo group (23.9 [15.2–28.5] % vs. 27 [21.9–31.9] %, *P* = 0.42). After excluding patients with no or minimal infarct, there was a trend toward reduction in infarct size in the Shenfu injection group (24.1 [20.3–29.3] % vs. 29.1 [24.5–32] %, *P* = 0.18). Incidence of adverse events was similar between the groups.

**Conclusions:** This pilot study showed that the use of Shenfu injection was safe but did not reduce infarct size by CMR Imaging and CK-MB release kinetics in reperfused patients with STEMI. Larger studies (confining to patients with extensive infarct size) to evaluate the efficacy of Shenfu injection on reperfusion injury are warranted.

**Clinical Trail Registration:** clinicaltrials.gov, identifier: NCT02709798.

## Introduction

The introduction of primary percutaneous coronary intervention (PCI) for ST-segment elevation myocardial infarction (STEMI) allows timely reperfusion for limiting infarct size and subsequent cardiac remodeling (1). However, reperfusion *per se* can trigger irreversible myocardial injury, a phenomenon called reperfusion injury. Many cardioprotective therapies have been proposed to reduce infarct size but have shown inconsistent clinical efficacy (2).

Shenfu injection is a traditional Chinese medicine formulation containing ginseng (Panax; family: Araliaceae) and aconite (Radix aconiti lateralis preparata, Aconitum carmichaeli Debx; family: Ranunculaceae), with ginsenosides and aconite alkaloids as the main active ingredients (3, 4). Its quality is strictly controlled in compliance with the standard of the China Ministry of Public Health (official approval code: certification number Z20043117; No. 110804, Ya'an, China) (3). Animal studies have shown that Shenfu injection has protective effects against reperfusion injury through multiple pharmacologic effects, such as scavenging free radicals, inhibiting inflammatory mediators, suppressing cell apoptosis, and inhibiting calcium overload (5–7). However, no data are available regarding its efficacy in patients with myocardial infarction. We aimed to determine whether the use of Shenfu injection, as compared to placebo, might reduce infarct size in patients with STEMI undergoing primary PCI.

## Methods

### Study Design and Participants

This was a single-center, randomized, blinded, placebo-controlled pilot study. From April 2016 to February 2018, patients aged 18 to 75 years with first-time anterior STEMI and scheduled for primary PCI within 6 h of symptom onset were eligible for enrollment. STEMI was diagnosed according to the third universal definition or myocardial infarction (8). An additional inclusion criterion was the presence of proximal or middle left anterior descending (LAD) occlusion with pre-PCI TIMI flow 0 or 1. Major exclusion criteria included patients with prior myocardial infarction and cardiogenic shock, and those receiving thrombolytic therapy. This study was conducted in accordance with the amended Declaration of Helsinki. The Institutional Review Board of Beijing Anzhen Hospital, Capital Medical University approved this study [D99-2015(043)-TCM-4]. All the patients provided written informed consent.

### Randomization, Intervention, and Outcomes

Eligible patients were randomized 1:1 to intravenous Shenfu injection (*n* = 20, 80 ml Shenfu injection + 70 ml 5% glucose injection) or matched placebo (*n* = 20, 150 ml 5% glucose injection) before reperfusion. The production process of Shenfu injection has been described elsewhere (9, 10). After informed consent was obtained, a blinded study medication box was opened. This box contained Shenfu injection or matched placebo (5% glucose injection) and labeled with a number that corresponded with the randomization list. This list was pre-specified as the treatment allocation list with serial numbers of 01 to 40 by Medical Statistics Office of Peking University First Hospital, Beijing, China. Randomization took place without stratification with a block size of 4. Treatment started ≥30 min before PCI and maintained for 5 days (once daily) after PCI (six times in total). Treatment assignment was blinded to both the patients and treating staff.

The primary end point was enzymatic infarct size assessed by creatine kinase-myocardial band (CK-MB) area under the curve (AUC) over 72 h (immediately after admission [0 h], and 6, 12, 18, 24, 48, and 72 h after PCI) and CMR Imaging (percent infarcted myocardium relative to left ventricular [LV] mass) 4 ± 1 days following PCI. Secondary end points included AUC for troponin I (TnI) over 72 h, peak value of CK-MB and TnI within 72 h, complete ST-segment resolution (≥70%) immediately post PCI, LV structure and function (left ventricular end diastolic volume [LVEDV], left ventricular end systolic volume [LVESV], and LV ejection fraction [LVEF]), and major adverse cardiovascular and cerebrovascular events (MACCE, such as death, non-fatal myocardial infarction, target vessel revascularization, stroke, new-onset heart failure during hospitalization, and re-hospitalization for heart failure) through 28 days post PCI. Details of methods, such as CMR Imaging protocol, are provided in Supplementary Material 1.

### Sample Size

Sample size calculation was limited considering that the primary outcome for Shenfu injection had not been previously evaluated at the time of the study design. Given the exploratory nature of this pilot study, a total of 40 participants were enrolled to collect preliminary data. After that, statistical analysis will be performed to assess whether the sample size meets the study design and a larger study may be performed.

### Statistical Analyses

Data analyses were performed according to the intention-to-treat concept. In addition, analyses were performed on a per protocol set, excluding major protocol violators. Continuous variables were presented as mean (SD) or median (first and third quartiles), and were compared by Student *t*-test or Wilcoxon rank sum test. Categorical variables were shown as the number (percentage), and were compared by Chi-square or Fisher exact test when appropriate. Wilcoxon rank sum test or CMH-χ2 test was performed for rank data. All the analyses were conducted with SAS version 9.4 (SAS Institute, Cary, NC, United States). All the tests were two-sided at a 5% significance level.

## Results

### Study Population and Clinical Characteristics

The flow of patient recruitment is shown in Supplementary Figure 1. Forty patients were randomized, and 32 (80%) completed CMR Imaging. Mean (SD) patient age was 54.4 (10.2) years, and 87.5% of the patients were men. The treatment groups were generally well-matched, although the mean age was younger in the Shenfu injection group (50.4 years) than in the placebo group (58.4 years). The total ischemic time and hemodynamic status (blood pressure, heart rate, and Killip class) were similar between the two groups. The use of stent, thrombus aspiration, glycoprotein IIb/IIIa inhibitors, and antiplatelet therapy was uniformly high in both arms (Table 1).

**Table 1:** Baseline characteristics by treatment assignment.

**Characteristic**	**Overall** **(*N* = 40)**	**Shenfu injection** **(*n* = 20)**	**Placebo** **(*n* = 20)**	***P*-value**
**Demographics**				
Age, mean (SD), y	54.4 (10.2)	50.4 (10.2)	58.4 (8.6)	0.01
Male	35 (87.5)	17 (85.0)	18 (90.0)	1.0
BMI, median (IQR), kg/m^2^	25.9 (23.3–27.0)	26.0 (23.0–27.7)	25.8 (23.3–26.7)	0.67
**Medical history**				
Diabetes	11 (27.5)	6 (30.0)	5 (25.0)	0.72
Hypertension	27 (67.5)	12 (60.0)	15 (75.0)	0.31
Hyperlipidemia	30 (75.0)	17 (85.0)	13 (65.0)	0.14
Current smoking	25 (62.5)	14 (70.0)	11 (55.0)	0.33
**Clinical presentation**				
Total ischemic time, mean (SD), min	276 (92)	274 (92)	278 (95)	0.91
Systolic BP, mean (SD), mmHg	137.6 (26.9)	141.3 (26.0)	134.0 (28.0)	0.40
Diastolic BP, mean (SD), mmHg	84.7 (20.2)	88.2 (20.0)	81.5 (20.6)	0.31
Heart rate, mean (SD), bpm	81.9 (13.2)	82.1 (11.7)	81.8 (14.8)	0.95
Killip class I	37 (92.5)	18 (90.0)	19 (95.0)	1.0
**Procedure**				
Diseased vessel				0.70
1 vessel	18 (45.0)	11 (55.0)	7 (35.0)	
2 vessels	12 (30.0)	3 (15.0)	9 (45.0)	
3 vessels	10 (25.0)	6 (30.0)	4 (20.0)	
PCI	37 (92.5)	18 (90.0)	19 (95.0)	1.0
Stenting	33 (82.5)	16 (80.0)	17 (85.0)	1.0
TIMI grade 3 post-PCI	37/37 (100%)	18/18 (100.0%)	19/19 (100.0%)	-
CTFC post-PCI, median (IQR), frame	11 (10–12)	11 (10–12)	11 (10–12)	0.85
Thrombus aspiration	28 (70.0)	15 (75.0)	13 (65.0)	0.49
Glycoprotein IIb/IIIa inhibitors	16 (40.0)	8 (40.0)	8 (40.0)	1.0
**Medication on discharge**				
Aspirin	38/39 (97.4)	20 (100.0)	18/19 (94.7)	0.49
Thienopyridine	39/39 (100.0)	20 (100.0)	19/19 (100.0)	–
Statin	38/39 (97.4)	19 (95.0)	19/19 (100.0)	1.0
β-blockers	37/39 (94.9)	19 (95.0)	18/19 (94.7)	1.0
ACEI/ARB	32/39 (82.1)	17 (85.0)	15/19 (78.9)	0.70

*ACEI, angiotensin-converting enzymes inhibitor; ARB, angiotensin receptor blocker; BMI, body mass index; BP, blood pressure; CTFC, corrected TIMI frame count; IQR, interquartile range; PCI, percutaneous coronary intervention; SD, standard deviation; TIMI, thrombolysis in myocardial infarction. Data are presented as mean (SD), median (IQR), n (%), or n/N (%)*.

### Efficacy Analysis

**Figure 1:**
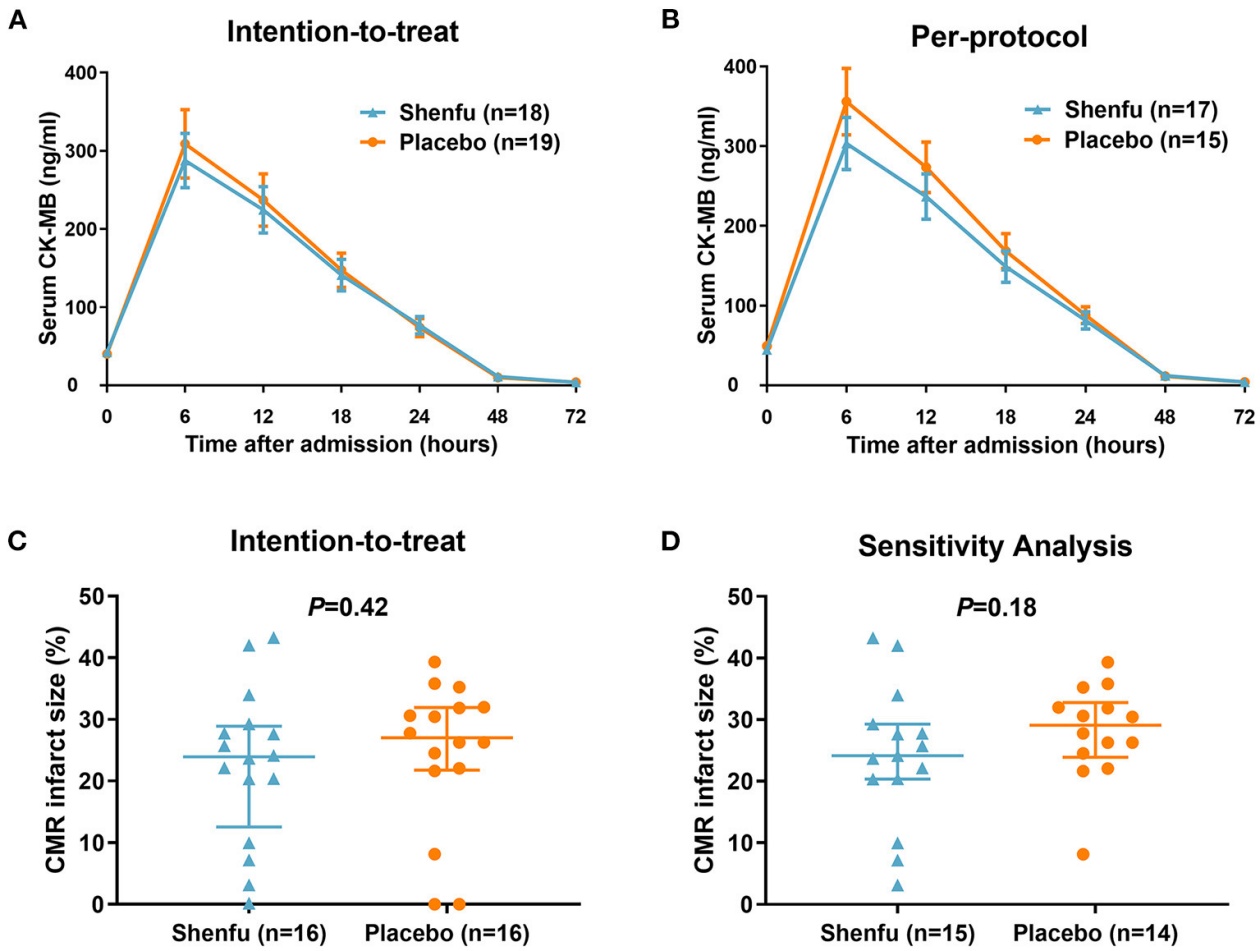
Primary outcomes by treatment assignment. **(A)** Intention-to-treat and **(B)** per-protocol analysis of infarct size by CK-MB area under the curve over 72 h post percutaneous coronary intervention, excluding patients with insufficient CK-MB values. **(C)** Intention-to-treat and **(D)** per-protocol analysis (sensitivity analysis by excluding patients with <1% infarct size) of CMR Imaging infarct size (relative to left ventricular mass) 4 ± 1 days following percutaneous coronary intervention. CK-MB, creatine kinase-myocardial band; CMR Imaging, cardiac magnetic resonance imaging.

Infarct size by AUC for CK-MB was not significantly decreased in the Shenfu injection group compared with the placebo group (intention-to-treat, 5602.5 [3539.4–7526.4] vs. 6403.2 [2234.4–8340.6] ng·h/ml, *P* = 0.82; per-protocol, 5902.2 [3960.3–7526.4] vs. 7561.8 [3840.9–8383.5] ng·h/ml, *P* = 0.27) (Figure 1). Likewise, the AUC for TnI was not significantly different in patients who received Shenfu injection compared with those who received the placebo (intention-to-treat, 2317 [1478.9–4498.4] vs. 2832.9 [738.2–6685.8] ng·h/ml, *P* = 0.96; per-protocol, 2372.9 [1623.4–4498.4] vs. 3608.3 [1200.6–6686] ng·h/ml, *P* = 0.39) (Supplementary Figure 2). There were no differences between the groups in peak CK-MB (295 [176.3–401] vs. 350.4 [102.2–494.6], *P* = 0.92) and TnI (117.5 [37.9–167] vs. 105 [33–210.6], *P* = 0.79) levels. The rate of complete ST-segment resolution (≥70%) was similar between the groups (36.8% vs. 38.9%, *P* = 0.9). Also, there were no differences between the groups in levels of high-sensitivity C-reactive protein 24 (227 [141–380] vs. 292.5 [147–452], *P* = 0.68) and 72 h (155 [64–237] vs. 180 [117–307], *P* = 0.72) after primary PCI.

Cardiac MRI was performed at a median of 4 (4 to 5) days after PCI. A nominal reduction in infarct size was observed in the Shenfu injection group compared with placebo group (23.9 [15.2–28.5] % vs. 27 [21.9–31.9] %, *P* = 0.42) (Figure 1 and Table 2). In the per-protocol analysis (sensitivity analysis), after excluding patients with no or minimal infarct (infarct size <1%), there was a trend toward reduction in infarct size in the Shenfu injection group (24.1 [20.3–29.3] % vs. 29.1 [24.5–32] %, *P* = 0.18) (Figure 1). The LV (LVEDV, LVESV, and LVEF) and other CMR Imaging parameters did not differ significantly between the groups (Table 2).

**Table 2:** Cardiac magnetic resonance imaging analysis 4+1 days post percutaneous coronary intervention.

**Characteristic**	**Overall** **(*N* = 40)**	**Shenfu injection** **(*n* = 20)**	**Placebo** **(*n* = 20)**	***P*-value**
CMR performed	32 (80.0)	16 (80.0)	16 (80.0)	1.0
CMR analyzed	32 (80.0)	16 (80.0)	16 (80.0)	1.0
CMR time from PCI, median (IQR), days	4 (4–5)	4 (4–5)	4 (4–5)	0.70
Infarct size^a^, median (IQR), %	26.0 (20.4–31.5)	23.9 (15.2–28.5)	27.0 (21.9–31.9)	0.42
LV mass, median (IQR), g	144.0 (124.0–182.5)	152.9 (131.2–177.4)	137.6 (120.2–189.6)	0.56
Area at risk (edema mass)^a^, median (IQR), %	41.7 (27.0–50.3)	39.9 (26.7–47.9)	47.9 (29.8–56.2)	0.36
Myocardial salvage index, median (IQR), %	41.9 (20.9–54.4)	40.4 (12.9–63.2)	41.9 (34.8–45.3)	0.98
MVO prevalence, %	24/32 (75.0)	12/16 (75.0)	12/16 (75.0)	1.0
MVO mass^a^, median (IQR), %	1.77 (0.01–3.02)	1.90 (0.01–3.11)	1.33 (0.07–2.96)	0.86
IMH prevalence, %	17/32 (53.1)	10/16 (62.5)	7/16 (43.8)	0.29
IMH mass^a^, median (IQR), %	0.31 (0.00–3.17)	1.08 (0.00–3.37)	0.00 (0.00–2.80)	0.45
LVEDV, mean (SD), ml	88.9 (27.4)	94.8 (27.5)	82.9 (26.8)	0.23
LVESV, mean (SD), ml	46.7 (23.0)	50.5 (23.8)	42.9 (22.3)	0.36
LVEF, mean (SD), %	49.6 (12.3)	48.7 (12.4)	50.5 (12.5)	0.69

*CMR, cardiac magnetic resonance; IMH, intramyocardial hemorrhage; IQR, interquartile range; LV, left ventricular; LVEDV, left ventricular end diastolic volume; LVEF, left ventricular ejection fraction; LVESV, left ventricular end systolic volume; MVO, microvascular obstruction; PCI, percutaneous coronary intervention; SD, standard deviation; TIMI, Thrombolysis In Myocardial Infarction. Data are presented as mean (SD), median (IQR), n (%), or n/N (%)*.

a *Expressed as a percentage of LV mass*.

### Clinical and Safety Outcomes

There was one ventricular fibrillation in the Shenfu injection group and one in the placebo group during primary PCI. One patient who received Shenfu injection underwent target vessel revascularization (coronary artery bypass graft) 22 days after PCI (non-treatment-related). Incidence of adverse events was similar between the groups (20 vs. 30%, *P* = 0.72) (Table 3). Moreover, Shenfu injection was not associated with an improvement in quality of life by the Myocardial Infarction Dimensional Assessment Scale (MIDAS) score at 28 days (60 [54–65] vs. 59 [48–63], *P* = 0.33).

**Table 3:** Safety outcomes.

**Events**	**Shenfu injection** **(*n* = 20)**	**Placebo** **(*n* = 20)**
**Severe adverse events**	0	0
**Total number of adverse events**	4	7
Fever	1	1
Nausea	0	1
Liver injury	1	0
Renal disorder	0	2
Urinary tract infection	1	0
Hypokalemia	0	2
Hyperhomocysteinemia	1	1
**Patients with at least 1 event**, ***n*** **(%)**	4 (20%)	6 (30%)

## Discussion

In this randomized trial of patients with first-time anterior STEMI undergoing primary PCI, the administration of Shenfu injection did not reduce infarct size, as assessed by CMR and CK-MB release kinetics. In the sensitivity analysis, by excluding patients with no or minimal infarct, there was a nominal 5% median reduction in CMR Imaging infarct size in the Shenfu injection group compared with placebo group.

Theoretically, the mechanisms underlying myocardial reperfusion injury are multifactorial, with multiple pathophysiological factors (such as oxidative stress, calcium overload, inflammation, and cell apoptosis) (11). A combining therapy to target different signaling pathways may provide more effective cardioprotection than a single targeted approach. Recently, the (12) NAC in Acute Myocardial Infarction (NACIAM) study demonstrated that the combined use of N-acetylcysteine and nitroglycerin before primary PCI reduced CMR Imaging-assessed infarct size by >30%.

As a Chinese herbal formula, Shenfu injection is characterized by multicomponents and multiple pharmacologic effects (13). In a recent multicenter randomized trial, Zhang et al. (9) demonstrated that Shenfu injection in combination with conventional post resuscitation care bundle treatment significantly improved survival in patients with return of spontaneous circulation after in-hospital cardiac arrest, probably by scavenging reactive oxygen species and inhibiting calcium overload and cardiomyocyte apoptosis (13). Animal studies have also confirmed the protective effects of Shenfu injection against ischemia-reperfusion injury through the aforementioned mechanisms (5–7).

In this study, we only enrolled patients with large anterior STEMI and within 6 h of onset with occlusion of the proximal/mid LAD, thereby representing a subset most likely to benefit from an adjunct therapy to primary PCI. Also, the drug infusion covered all the reperfusion phases (30 min before PCI and up to 5 days after PCI) to ensure adequate therapeutic levels. Although the primary analysis was non-significant, in patients with extensive infarct (excluding patients with no or minimal infarct), there was a trend toward reduction in CMR Imaging infarct size in the Shenfu injection group compared with placebo group. The results of our study need to be validated in larger trials (particularly confining to patients with estimated larger infarct size).

### Study Limitations

Our study has some limitations. First, the sample size was small. Thus, the power to examine the efficacy of intervention was limited. Second, 20% of the patients did not undergo CMR Imaging for primary outcome analysis. Third, we evaluated only a single dose of Shenfu injection and cannot exclude different results with varying dose regimens.

## Conclusions

In this pilot study on patients with first-time anterior STEMI undergoing primary PCI, treatment with Shenfu injection was safe but was not associated with reduction in infarct size as assessed by CMR Imaging and CK-MB release kinetics. Larger studies (confining to patients with extensive infarct size) to evaluate the efficacy of Shenfu injection on reperfusion injury are warranted.

## Data Availability Statement

The raw data supporting the conclusions of this article will be made available by the authors, without undue reservation.

## Ethics Statement

The studies involving human participants were reviewed and approved by Institutional Review Board of Beijing Anzhen Hospital, Capital Medical University. The patients/participants provided their written informed consent to participate in this study.

## Author Contributions

XW and SN: conception, design, data analysis, and interpretation. XM and SN: administrative support. XW, HM, YY, and SN: provision on study materials or patients. XW, HM, YY, RG, WG, YH, and HW: collection and assembly of data. All authors manuscript writing and final approval of the manuscript.

## Funding

This study was funded in part by the National Natural Science Foundation of China (81870322), Beijing Municipal Administration of Hospitals' Ascent Plan (DFL20180601), Natural Science Foundation of Beijing, China (7191002), Beijing Nova Program (Z201100006820087), Fund for Beijing Science & Technology Development of TCM (QN2018-01), and Beijing Municipal Administration of Hospitals Incubating Program (PZ2019005).

## Conflict of Interest

SN research grants to the institution from Boston Scientific, Abbott, Jiangsu Hengrui Pharmaceuticals, China Resources Sanjiu Medical & Pharmaceuticals, East China Pharmaceuticals. The remaining authors declare that the research was conducted in the absence of any commercial or financial relationships that could be construed as a potential conflict of interest.

## Publisher's Note

All claims expressed in this article are solely those of the authors and do not necessarily represent those of their affiliated organizations, or those of the publisher, the editors and the reviewers. Any product that may be evaluated in this article, or claim that may be made by its manufacturer, is not guaranteed or endorsed by the publisher.

## References

[1] Heusch G Libby P Gersh B Yellon D Böhm M Lopaschuk G. Cardiovascular remodelling in coronary artery disease and heart failure. Lancet. (2014) 383:1933–43. 10.1016/S0140-6736(14)60107-0 24831770 PMC4330973

[2] Hausenloy DJ Yellon DM. Combination therapy to target reperfusion injury after ST-segment-elevation myocardial infarction: a more effective approach to cardioprotection. Circulation. (2017) 136:904–6. 10.1161/CIRCULATIONAHA.117.029859 28874421

[3] Song Y Zhang N Shi S Li J Zhang Q Zhao Y. Large-scale qualitative and quantitative characterization of components in Shenfu injection by integrating hydrophilic interaction chromatography, reversed phase liquid chromatography, and tandem mass spectrometry. J Chromatogr A. (2015) 1407:106–18. 10.1016/j.chroma.2015.06.041 26143607

[4] Yang H Liu L Gao W Liu K Qi LW Li P. Direct and comprehensive analysis of ginsenosides and diterpene alkaloids in Shenfu injection by combinatory liquid chromatography-mass spectrometric techniques. J Pharm Biomed Anal. (2014) 92:13–21. 10.1016/j.jpba.2013.12.041 24469096

[5] Zheng SY Sun J Zhao X Xu JG. Protective effect of shen-fu on myocardial ischemia-reperfusion injury in rats. Am J Chin Med. (2004) 32:209–20. 10.1142/S0192415X04001874 15315259

[6] Wu Y Xia ZY Meng QT Zhu J Lei S Xu J. Shen-Fu injection preconditioning inhibits myocardial ischemia-reperfusion injury in diabetic rats: activation of eNOS via the PI3K/Akt pathway. J Biomed Biotechnol. (2011) 2011:384627. 10.1155/2011/384627 21151615 PMC2997576

[7] Wang YY Li YY Li L Yang DL Zhou K Li YH. Protective effects of Shenfu injection against myocardial ischemia-reperfusion injury via activation of eNOS in rats. Biol Pharm Bull. (2018) 41:1406–13. 10.1248/bpb.b18-00212 29910216

[8] Thygesen K Alpert JS Jaffe AS Simoons ML Chaitman BR White HD. Third universal definition of myocardial infarction. Eur Heart J. (2012) 33:2551–67. 10.1016/j.gheart.2012.08.001 22922414

[9] Zhang Q Li C Shao F Zhao L Wang M Fang Y. Efficacy and Safety of combination therapy of Shenfu injection and postresuscitation bundle in patients with return of spontaneous circulation after in-hospital cardiac arrest: a randomized, assessor-blinded, controlled trial. Crit Care Med. (2017) 45:1587–95. 10.1097/CCM.0000000000002570 28661970

[10] Zhu J Kang L Ye Q Fan G Liang Y Yan C. Effects of Shenfu injection and its main components on the contraction of isolated rat thoracic aortic rings. PLoS ONE. (2013) 8:e78026. 10.1371/journal.pone.0078026 24205074 PMC3813522

[11] Hausenloy DJ Botker HE Engstrom T Erlinge D Heusch G Ibanez B. Targeting reperfusion injury in patients with ST-segment elevation myocardial infarction: trials and tribulations. Eur Heart J. (2017) 38:935–41. 10.1093/eurheartj/ehw145 27118196 PMC5381598

[12] Pasupathy S Tavella R Grover S Raman B Procter NE Du YT. Early use of N-acetylcysteine with nitrate therapy in patients undergoing primary percutaneous coronary intervention for st-segment-elevation myocardial infarction reduces myocardial infarct size (the NACIAM Trial [N-acetylcysteine in acute myocardial infarction]). Circulation. (2017) 136:894–903. 10.1161/CIRCULATIONAHA.117.027575 28634219

[13] Zhang Q Li C. The roles of traditional chinese medicine: shen-fu injection on the postresuscitation care bundle. Evid Based Complement Alternat Med. (2013) 2013:319092. 10.1155/2013/319092 24066009 PMC3771486

