# Supplementary material for: Effect of Shenfu Injection on Reperfusion Injury in Patients Undergoing Primary Percutaneous Coronary Intervention for ST Segment Elevation Myocardial Infarction: A Pilot Randomized Clinical Trial
Source: Front Cardiovasc Med. 2021 Dec 3;8:736526. doi: 10.3389/fcvm.2021.736526 (PMC8678404; doi:10.3389/fcvm.2021.736526)
Supplement: Supplementary file 1 [file Data_Sheet_1.DOCX]

**Effect of Shenfu Injection on Reperfusion Injury in Patients Undergoing Primary Percutaneous Coronary Intervention for ST segment Elevation Myocardial Infarction: A Pilot Randomized Clinical Trial**

**Supplemental Methods**

**CMR Protocol and Analysis**

Cardiac magnetic resonance (CMR) scanning was performed with a 3.0-Tesla system (Magnetom Verio, Siemens AG Healthcare, Erlangen, Germany) at 4±1 days after reperfusion. All sequences were acquired in breathhold. Cine imaging was acquired by a steady-state free precession sequence in short-axis view covering the whole left ventricle (LV) without gap (repetition time/echo time, TR/TE 3.5/1.5 ms, 25 phases, voxel size 2.0×1.6×8 mm^3^). Black blood T2-weighted short tau inversion-recovery (T2w-STIR) images were acquired at apical, mid-ventricle, and basal level on short-axis plane (TR/TE 2×R-R intervals/75 ms, voxel size 2.0×1.6×8 mm^3^). Late gadolinium enhancement (LGE) images were acquired with an inversion recovery segmented 3D gradient echo sequence 10 minutes after contrast injection (0.2mmol/kg; Magnevist, Bayer HealthCare Pharmaceuticals Inc., Germany) at short-axis and 2-, 4-chamber views (TR/TE 3.5/1.7 ms, temporal resolution 190 ms, voxel size 1.5×1.7×10 mm^3^ interpolated into 0.74×0.74×5 mm^3^) ^1^.

CMR analysis was performed offline in a core lab (Department of Cardiology, Renji Hospital, School of Medicine, Shanghai Jiaotong University) using CVI42 Version 5.6.1 (Cardiovascular Imaging, Calgary, Alberta, Canada) by 2 independent physicians, who were blinded to all patient details and treatment allocation. LV volumes and mass were calculated from cine images as previously described ^1^. Infarction was defined as hyperenhanced myocardium with signal intensity of >5 standard deviations (SDs) of remote normal myocardium and quantified as a percentage of LV mass. Microvascular obstruction (MVO) was defined as any hypoenhanced area within infraction. Edema (area at risk, AAR) was quantified as hyperenhancement on T2w-STIR imaging within the territory of culprit vessel (signal intensity >2 SDs above the mean signal in remote skeletal muscle) and intramyocardial hemorrhage (IMH) was recognized as hypoenhanced area within the edema. Myocardial salvage index (MSI) was calculated as the percentage of the AAR that was not infarcted on LGE images: (AAR−infarct size/AAR)×100%.

**Interobserver and Intraobserver Variability**

Ten CMR images were randomly selected and analyzed for infarct size and AAR by 2 independent readers (YH, HW) and by the same reader (HW) after 4 weeks. Interobserver and intraobserver reproducibility of image analysis were assessed by intraclass correlation coefficients (ICC) for continuous variable.

**Supplemental Results**

**Interobserver and Intraobserver Variability**

ICC for interobserver agreement was 0.87 and 0.89 (for infarct size and AAR respectively) and for intraobserver variability was 0.85 and 0.89 (for infarct size and AAR respectively).

**Reference**

1. Ding S, Li Z, Ge H, et al. Impact of Early ST-Segment Changes on Cardiac Magnetic Resonance-Verified Intramyocardial Haemorrhage and Microvascular Obstruction in ST-Elevation Myocardial Infarction Patients. Medicine (Baltimore). 2015;94(35):e1438.

**Supplemental Figure 1.** **Flow Diagram.**


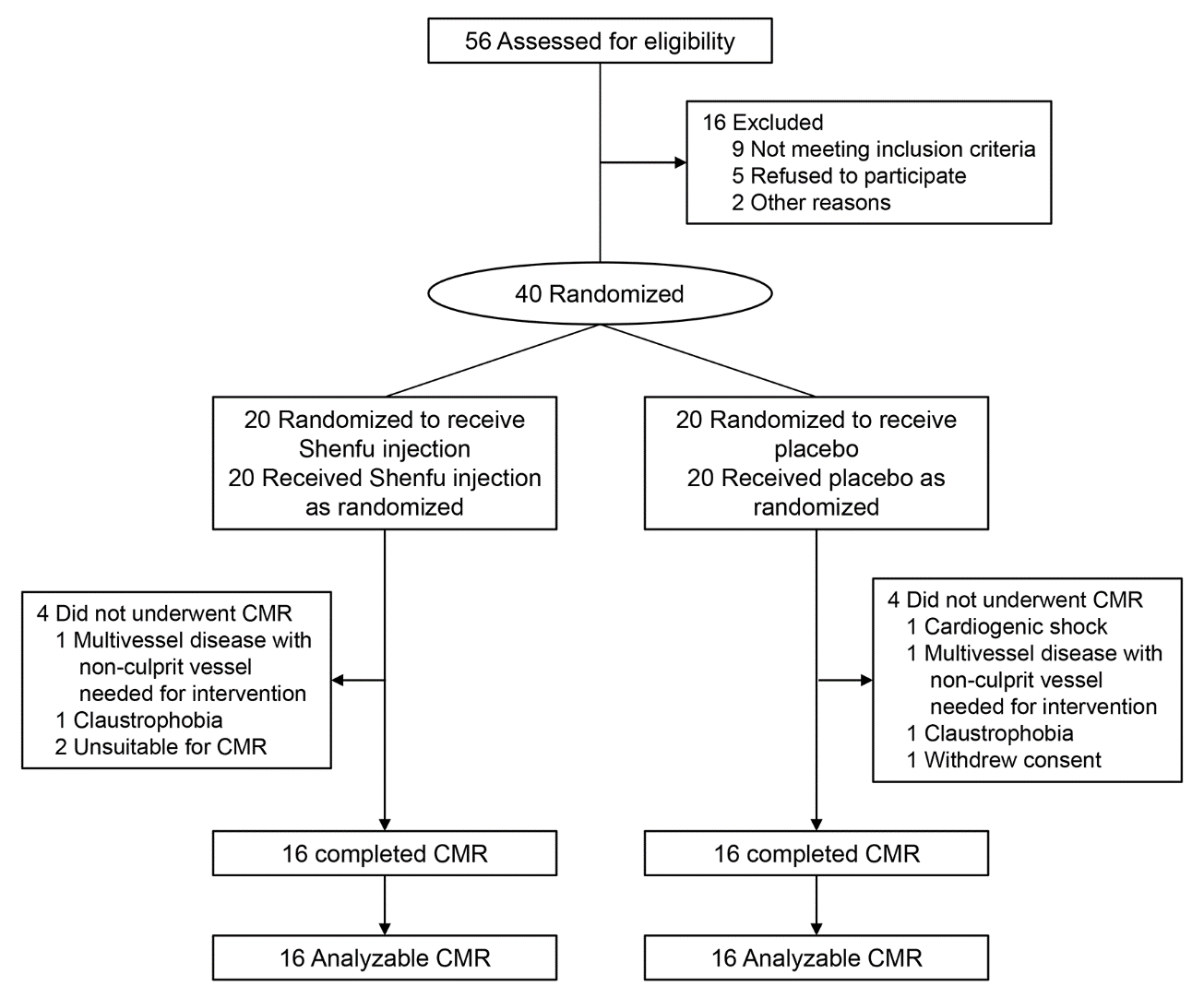


Abbreviations: CMR, cardiac magnetic resonance.

**Supplemental Figure 2. Secondary Outcome of Troponin I Kinetics by Treatment Assignment.**

**
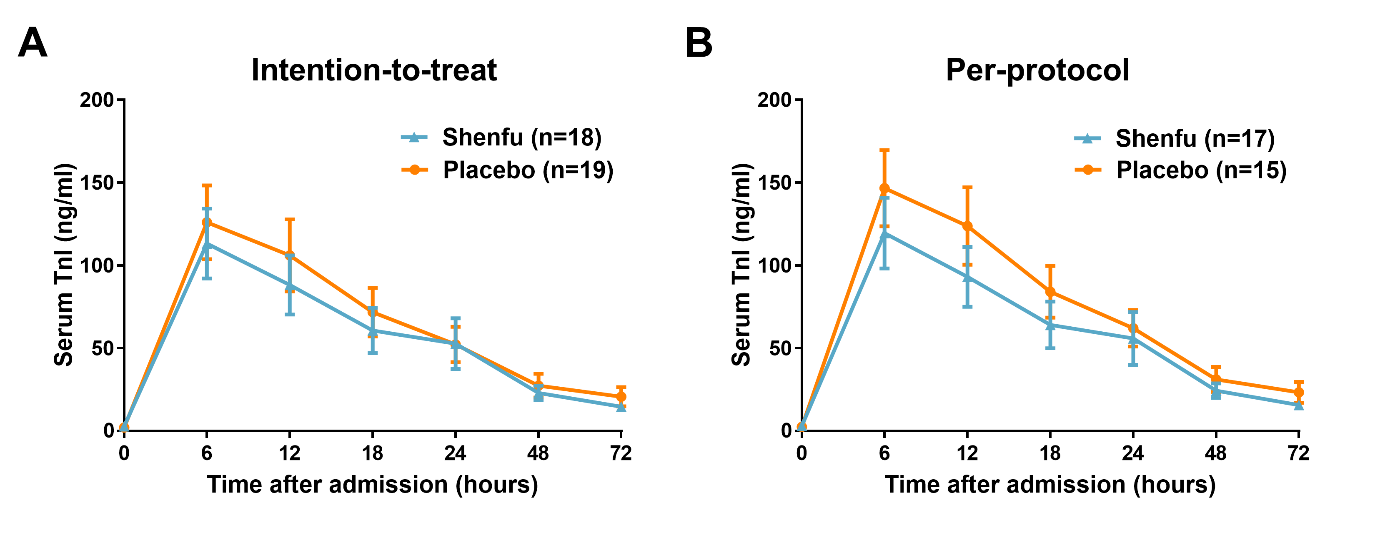
**

**Figure Legend.**

Intention-to-treat (A) and per-protocol analysis (B) of infarct size by TnI area under the curve over 72 h post percutaneous coronary intervention, excluding patients with insufficient TnI values. Abbreviations: TnI, troponin I.
